# Supplementary material for: Conversion of Retinyl Palmitate to Retinol by Wheat Bran Endogenous Lipase Reduces Vitamin A Stability
Source: Foods. 2023 Dec 25;13(1):80. doi: 10.3390/foods13010080 (PMC10778787; doi:10.3390/foods13010080)
Supplement: Supplementary file 1 [file foods-13-00080-s001.zip › foods-2720105-supplementary.pdf]

Supplementary material

1. Composition of wheat bran and soy oil

Table S1: Approximate composition of the wheat bran used in this study as supplied by the supplier.

Component	Content (%)
Carbohydrates	12
Fibres	50
Proteins	17
Lipids	5
Ash	7
Moisture	9

Table S2: Fatty acid content (g/100 g oil) (mean ± standard deviation) of the soy oil used in this study, measured by analysis of fatty acid methyl esters using gas chromatography with flame ionization detection.

Fatty acid	Content (g/100 g oil)
Caprylic acid (C10:0)	0.16 ± 0.01
Myristic acid (C14:0)	0.12 ± 0.01
Palmitic acid (C16:0)	7.84 ± 0.88
Stearic acid (C18:0)	2.86 ± 0.33
Oleic acid (C18:1)	20.62 ± 2.36
Linoleic acid (C18:2)	36.30 ± 1.91
α-linolenic acid (C18:3, ω-3)	4.39 ± 0.59
Arachidic acid (C20:0)	0.27 ± 0.03
Eicosenoic acid (C20:1)	0.15 ± 0.02
Behenic acid (C22:0)	0.30 ± 0.06
Tricosylic acid (C23:0)	0.00 ± 0.00
Docosahexaenoic acid (C22:6, ω-3)/ Lignoceric acid (C24:0)	0.12 ± 0.02
Nervonic acid (C24:1)	0.16 ± 0.01

2. Characterisation of retinyl tridecanoate

<u>Elemental analysis</u>

The elemental composition (C, H, N and S) of retinyl tridecanoate (RTD) was determined using a Thermo Scientific FLASH 2000 elemental analyser (Eyley and Thielemans, 2014). A calibration curve was created with 2,5-Bis(5-*tert*-butyl-2-benzo-oxazol-2yl)thiophene (BBOT) as standard and vanadium (V) oxide for accurate sulphur determination. Elemental analysis (EA) results are shown in Table S3. The analysis was performed on 1.07 mg RTD.

Table S3: Elemental composition of RTD, experimentally determined by EA and theoretically calculated based on the chemical formula $C_{33}H_{54}O_2$.

	O (wt%)	C (wt%)	H (wt%)	N (wt%)	S (wt%)
Experimental	-	76.95	10.88	0.00	0.00
Calculated	6.63	82.10	11.27	-	-

Deviations between the experimental and calculated values can be explained by the excess tridecanoyl chloride used for the synthesis. The resulting tridecanoic acid has relative lower amounts of carbon and hydrogen, while oxygen presence is higher.

The absence of nitrogen in the results suggests that impurities from the used base, DIPEA, were removed from the sample by washing.

Fourier transform infrared spectroscopy

A Bruker ALPHA Fourier transfor infrared (FT-IR) spectrophotometer was used to determine the presence of an ester bond, confirming the reaction. A droplet of dissolved RTD (0.5 mg/mL) was placed on the attenuated total reflectance (ATR) sample surface and measured after the solvent was evaporated. The spectrum was calculated as the sum of 24 scans, measured between 4000 and 400 cm^{-1}. The spectrum is shown in Figure S1 with the identification of the major peaks in Table S4.

Figure S1: ATR-FT-IR spectrum of RTD.

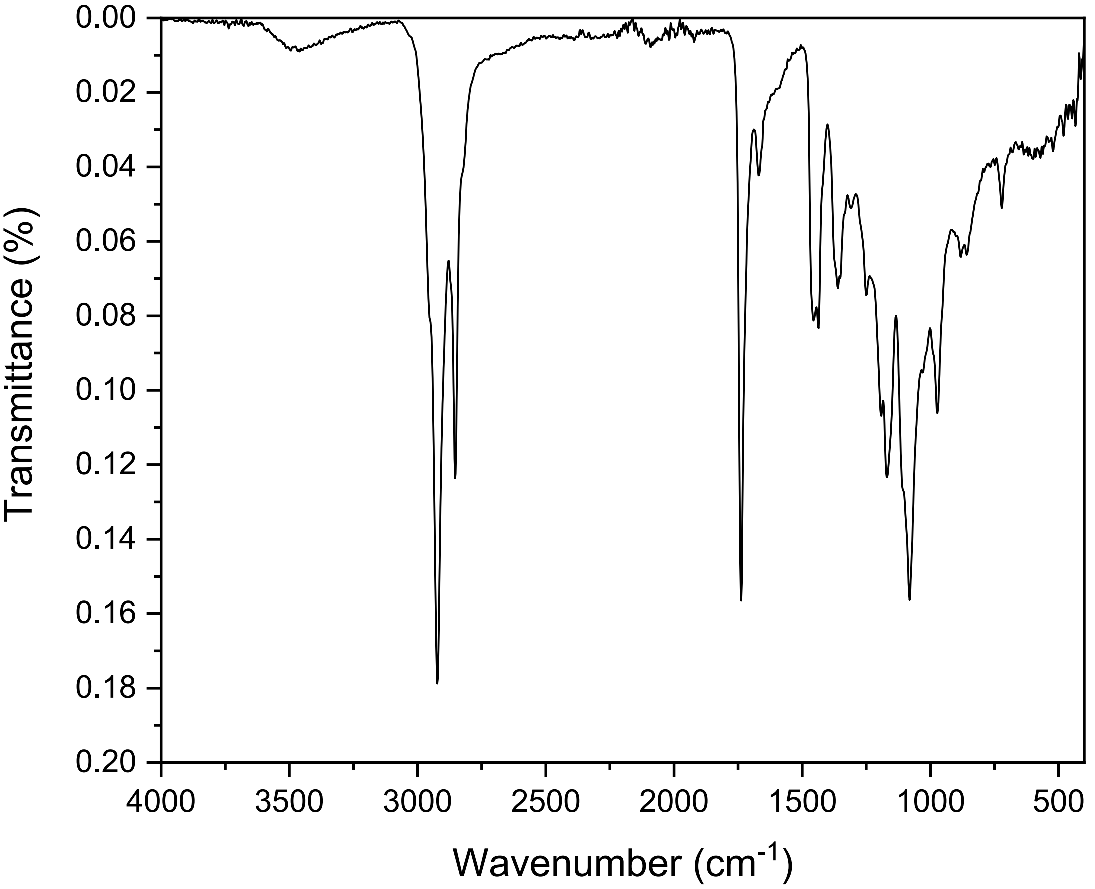

Table S4: Data table for the most prominent ATR-FT-IR peaks for RTD.

**Bond**	Wavenumber (cm^{-1})
**v (C-H)**	2922.46
**v (C-H)**	2852.85
**v (C-H) / v (C=C)**	1738.18
**v (C=O)**	1669.56
**δ (C-O-H)**	1436.73
**δ (C-O-H)**	1360.76
**v (C-O-C)**	1169.62
**δ (C-O-C)**	1081.24
ω (C=C)	972.84

The peak present at 1669.56 cm^{-1} indicates the formation of an ester bond. This peak is not present in FTIR spectra of retinol or tridecanoic acid (Kim et al., 2016; Marikhin et al., 2017).

Reference list

Eyley, S. and Thielemans, W. (2014). Surface modification of cellulose nanocrystals. *Nanoscale*, *6*(14), 7764–7779. https://doi.org/10.1039/c4nr01756k

Kim, H., Hu, Y., Jeong, D., Jun, B. H., Cho, E. and Jung, S. (2016). Synthesis, characterization, and retinol stabilization of fatty amide-β-cyclodextrin conjugates. *Molecules*, *21*(7). https://doi.org/10.3390/molecules21070963

Marikhin, V. A., Myasnikova, L. P., Radovanova, E. I., Volchek, B. Z. and Medvedeva, D. A. (2017). Fourier transform infrared spectroscopic study of the kinetics of a first-order phase transition in tridecanoic acid CH3(CH2)11COOH. *Physics of the Solid State*, *59*(2), 331–337. https://doi.org/10.1134/S1063783417020184
